# The Jigsaw teaching method compared to traditional teaching on anatomy and physiology knowledge in higher education – a randomised controlled trial

**DOI:** 10.1186/s12909-026-08608-x

**Published:** 2026-01-17

**Authors:** Janette Moland Stokstad, Eirik Solberg Nedrehagen, Karl Ove Hufthammer

**Affiliations:** 1 Department of Welfare and Participation, Western Norway University of Applied Sciences, Sogndal, Norway; 2 Department of Welfare and Participation, Western Norway University of Applied Sciences, Bergen, Norway

**Keywords:** Jigsaw teaching method, Student-centred learning, Anatomy and physiology education, Learning outcomes, Student perceptions

## Abstract

**Background:**

Traditional teaching (TT) is lecturer-centred, while student-centred teaching, including Jigsaw (JS), fosters student interaction. However, the results of research on the effectiveness of JS on learning outcomes is inconsistent. This randomised controlled trial compared the effect of JS and TT on higher-education students’ knowledge of the anatomy and physiology of the sensory apparatus and on their perceptions of the two teaching methods.

**Methods:**

Forty-eight undergraduate social nursing students were randomised to either a TT or JS-based learning activity. One JS student dropped out before completing the activity and was excluded, resulting in 47 participants (23 TT, 24 JS). Improvement in knowledge was assessed by recording the number of correct answers (*points*) on a 25-question multiple-choice test at three time points: before the activity (*baseline*), immediately after the activity (*post-intervention*) and at a three-month follow-up. Additionally, the students’ perceptions were surveyed post-intervention using a questionnaire. Improvement in knowledge was analysed using a longitudinal model, and group differences in perceptions were analysed using Wilcoxon–Mann–Whitney tests.

**Results:**

Both groups significantly improved knowledge from baseline to post-intervention (TT: 5.2 points; JS: 4.6 points; *p* < 0.001 both groups) and from baseline to the three-month follow-up (TT: 3.8 points; JS: 3.3 points; *p* < 0.001 for both groups). No statistically significant differences in knowledge were observed between the groups (post-intervention difference: −0.5 points, 95% CI: −2.3 to 1.2, *p* = 0.54; three-month difference: −0.6 points, 95% CI: −2.4 to 1.2, *p* = 0.52). Overall, 91% improved from baseline to post-intervention, and 83% from baseline to three months. A higher proportion of TT students than JS students preferred their assigned teaching activity over others (78% vs. 38%, *p* = 0.002) or believed it would improve their grades (87% vs. 42%, *p* = 0.004). On the other hand, a *lower* proportion thought that it provided useful practice in oral presentation / communication (61% vs. 88%, *p* = 0.02).

**Conclusions:**

This study found that both the Jigsaw method and traditional lectures effectively improve anatomy and physiology knowledge among students. Despite a student preference for traditional teaching, there were no significant differences in knowledge acquisition or retention between the two methods.

**Trial registration:**

Clinical trial number not applicable.

## Background

Since the emergence of universities in medieval Europe, traditional teaching (TT) with didactic lectures has been the dominating form of pedagogy in higher education [1]. In the context of anatomy teaching, a still ongoing, two-decade-long transition has gradually shifted TT towards more active, student-centred learning [2, 3], . Student-centred learning (SCL), a more active form of learning, has gradually been integrated into traditional university curricula [4]. In active learning, the student take ‘an active role in their learning process through activities such as group discussions, problem solving, and applied activities’ [5]. It promotes cooperation and interaction between students, encourages a deeper understanding of the subject [6, 7], may improve academic results [7] and can possibly be more effective compared to teaching with the student as a passive receiver [8–10].

In the context of teaching anatomy and physiology, SCL can enhance learning by aligning the content to be taught, the pedagogy and the standards expected upon graduation [11]. It can involve various approaches, such as the flipped classroom, problem-based learning and the Jigsaw teaching method (JS) [12]. In anatomy and physiology teaching, JS can be particularly suited, as it may enable students to learn collaboratively, articulate explanations for themselves and others, and actively construct meaning [10]. It also gives students an opportunity to learn factual knowledge (‘knowing what’) in a shared, professional field relevant to their future career. This knowledge then provides a foundation for developing practical skills later (‘knowing how’) [10].

The JS method was developed by Arnason and colleagues in the 1970s [13]. It works like this: A central topic is first divided into sub-topics. Students are initially organised into groups where each member is assigned a unique sub-topic to study. Thereafter, the students are further reorganised into expert groups, where each expert group focuses on a specific sub-topic. After deepening their understanding within the expert groups, students return to their initial group to teach their respective sub-topic to their peers. Through the peer teaching, the students collectively cover the full topic in their initial groups [13, 14].

The JS method is one of the most extensively studied forms of SCL across diverse academic contexts, and its impact on learning appears to be context-dependent [15]. Research across different educational levels and settings shows inconsistent and heterogeneous effects on learning achievement [15].

Within the specific context of anatomy and physiology teaching in health and medical education, several studies have investigated the effect of JS. Two studies reported improvements in anatomy knowledge, one using a non-controlled design and non-identical pre–post multiple-choice tests (MCTs) [16], and the other using a non-randomised controlled design and identical pre–post MCTs as well as end-of- semester exam scores [17].

Two additional studies compared the effects of JS and TT on nursing students’ knowledge of anatomy and physiology. One employed a randomised quasi-experimental design [18] while the other used a randomised crossover design [19]. Both studies demonstrated that JS was more effective than TT in the short-term, and Chandel et al. [19] further reported that JS also may be more effective for retaining knowledge in the long-term (one month).

All these studies assessed learning effects over time at the *group* level, reporting on average outcomes, without examining and presenting data at the *individual* level. This limits our understanding of how JS may impact students differently.

Multiple studies have also examined students’ *perceptions* of JS [14, 16, 17, 19, 20]. And while one study has compared students’ perceptions of JS and self-directed learning [21], no study has compared their perceptions of JS to those of TT.

Given the limited number of comparative studies using randomised controlled designs and the lack of research comparing students’ perceptions of JS and TT, there is a need for more robust evidence to inform future development of teaching procedures.

## Methods

### Research questions and study design

The aim of this study was to answer the following research questions: When teaching anatomy and physiology using the Jigsaw method or traditional teaching, what are.

1. the *overall* (average) short- and long-term effects on students’ knowledge,
2. the *individual* short- and long-term effects on students’ knowledge,
3. students’ perceptions of the teaching methods,

and how do these effects and perceptions differ between students exposed to Jigsaw or to traditional teaching?

To answer these research questions, we conducted a randomised controlled trial. Students were randomly allocated to either teaching using JS or TT. We assessed the students’ knowledge immediately before and after the teaching intervention and at a three-month follow-up. We also collected a post-intervention student evaluation of the teaching.

### Participants and organisation of the teaching activities

A total of 95 (61 full-time and 34 part-time) secondary-year undergraduate students in social nursing at the University of Western Norway were invited to participate in the study. They received information about the study both orally and in writing 30 days prior to the intervention.

**Fig. 1:**
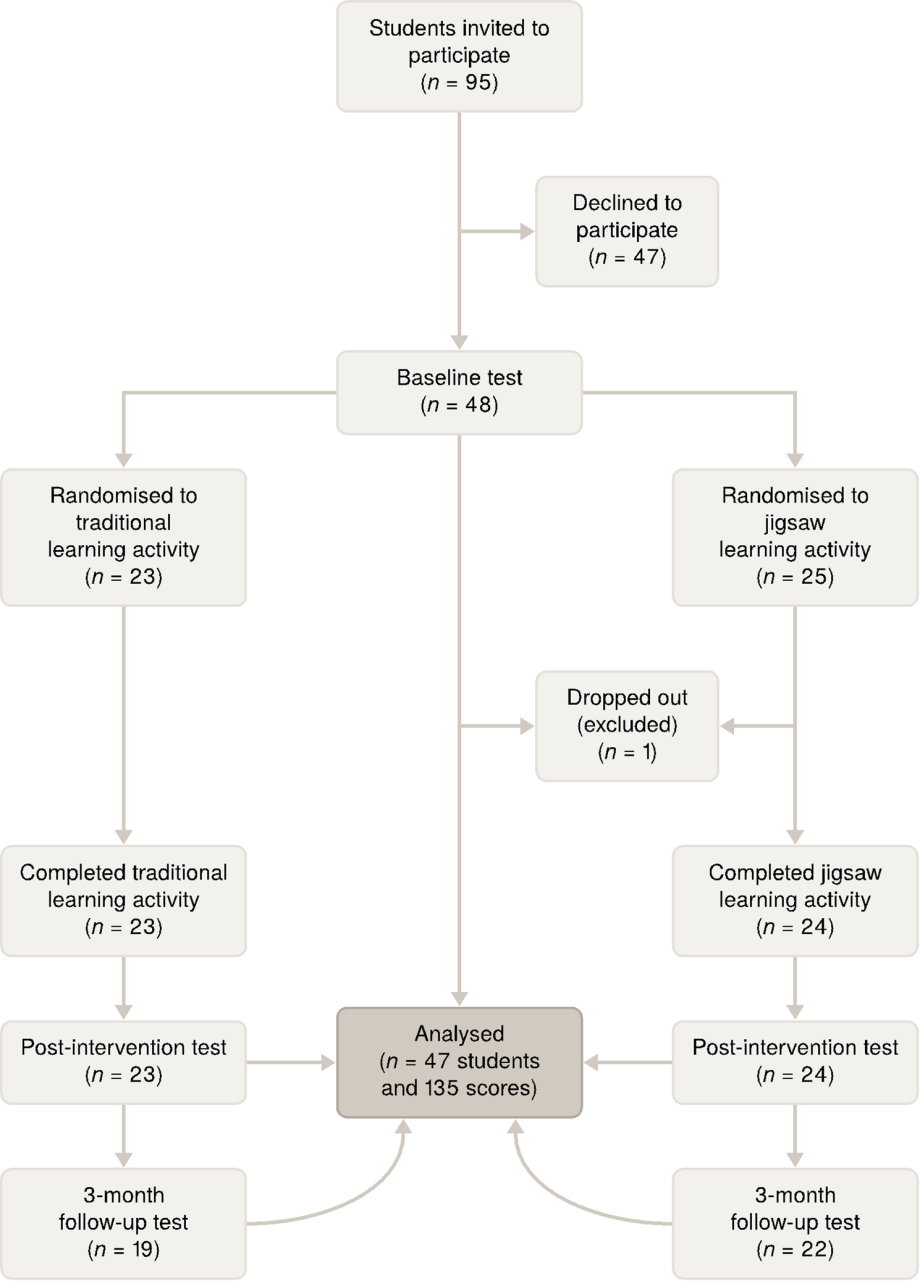
Study flow chart

Of all invited students, 48 (26 full-time and 22 part-time) agreed to participate and signed an informed consent form. One participant later dropped out, leaving 47 to complete the study (Fig. 1). Their mean age was 30 years (SD 9, range 19–52), and 74% were female. The participants had an average of 7 years (SD 9, range 0–28) of previous work experience, and most (60%) had no previous higher education.

All participants had previously attended both TT and SCL during the course, but this was the first JS session in the current educational programme. As the teaching activity was conducted midway through the anatomy and physiology course, participants had the opportunity to attend other classes in advance, covering topics such as the cell, nervous system, digestive system, circulatory system and respiratory system.

On the day of the teaching activity, all participants were collectively informed about the study procedures and were presented with the underlying curriculum and learning objectives as outlined in the course description: *to acquire knowledge about the human physiology*,* anatomy*,* basic needs and development from a life-course perspective.* They were further informed that the teaching would address the following curriculum sub-topics: (1) sense of sight; (2) sense of hearing; (3) smell and proprioception; (4) sense of touch, including pain; (5) taste and equilibrioception. Subsequently, the participants received specific learning instructions: *describe and explain the anatomical structure and function of central elements.* A detailed schedule is provided in Table 1.

**Table 1:** Experiment schedule

Time	JS	TT
09:00–09:30	Study information	Study information
09:30–10:00	Baseline MCT	Baseline MCT
10:15–12:00	Work in expert groups	Traditional teaching
12:00–12:30	Teaching in primary groups	Post-MCT and questionnaire
12:30–13:00	Teaching in primary groups	
13:00–13:30	Post-MCT and questionnaire	

The study was conducted in two consecutive tracks: one for full-time students and one for part-time students, both implemented in accordance with the described study procedures. Full-time students complete a three-year programme with primarily on-campus teaching, whereas part-time students follow a four-year programme using a blended model of weekly online sessions and monthly, week-long campus gatherings. Apart from this distinction, full-time and part-time students receive an equivalent amount of teaching activities and coverage of the curriculum, and the curriculum is the same for the full-time and part-time track.

### Test and questionnaire design

A curriculum-based MCT was systematically developed by two of the authors in line with Kanzow et al. [22], Krathwohl [23] and Considine et al. [24]. The test consisted of 25 questions of medium difficulty, organised into five categories reflecting the curriculum sub-topics. Within each category, three questions targeted factual knowledge, while two questions targeted conceptual understanding, in alignment with Bloom’s Taxonomy [23, 25]. Each question had three plausible alternative answers of approximately the same length [25, 26].

To ensure face and content validity as described by Brenner et al. [25], the MCT was reviewed and evaluated independently by five anatomy and physiology lecturers at the Western Norway University of applied sciences. They provided feedback and suggested revisions to the preliminary version. The suggested edits included adjustments to the level of difficulty in two questions, overall linguistic improvements and terminology changes. We made changes to language and question difficulty in accordance with the feedback. However, terminology was not altered, as the proposed changes did not align with the terminology in the course curriculum.

A questionnaire adapted from Oakes et al. [17] was used to evaluate perceived experiences of the teaching methods. The questionnaire consisted of seven four-point Likert items measuring agreement (‘strongly disagree’, ‘somewhat disagree’, ‘somewhat agree’, ‘strongly agree’), and was translated into Norwegian and subsequently modified by the authors to suit the specific aim of this study. The translated version was reviewed and evaluated by five university lecturers in anatomy and physiology and piloted with a sample of 25 s-year social nursing students.

### Assessment of MCT performance

The MCT total performance was measured as a sum score, where each correct answer was awarded one point and each incorrect answer, zero points [22]. No points were awarded if multiple answers were selected for a single question or if a question was left unanswered. The maximum possible score was 25. All responses on the questionnaire and the MCT sum score were independently assessed by two assessors. If a conflict occurred, the assessors sought mutual agreement.

### Randomisation

Following the pre-test, participants were randomly assigned into two groups using Microsoft Excel for Microsoft 365. The randomisation procedure was carried out on site by one of the authors. Participants were assigned a random number using the = RAND( ) function, and the participant list was subsequently sorted from lowest to highest based on this random number. As the JS method required groups of five students, the first 15 full-time and first 10 part-time students in the participant lists were allocated to the JS group (*n* = 25), while the remaining participants on the list were allocated to the TT group (*n* = 23). Due to the educational nature of the intervention, it was not possible to blind either the lecturer or the students. The students were not aware of which group they were randomised to until minutes before the teaching began. The JS group remained in the classroom for further randomisation and group work, while the TT group was relocated to a new classroom in a different building. A lecturer was constantly present with the students throughout the teaching procedures.

### Test administration

The pre-test was administered to all the participants simultaneously immediately after the briefing on the study procedures (Table 1) and was overseen by two lecturers. All participants were informed that each question had only one correct answer. They were instructed to complete the test with pen and paper, relying entirely on their knowledge and without the use of aids such as the internet or course literature. It was emphasised that the results on the MCT would not influence the final course grades. In accordance with the ‘one minute rule’ [27], the time limit for the test was 25 min. Participants were evenly spread out in the classroom with > 1 m distance during the test administration.

An identical post-test was administered to the JS and TT group immediately after the corresponding teaching session ended, in their respective classrooms, and was overseen by the lecturer. Background variables (sex, age, years of full-time studies before social-nursing education, years with full-time work experience) were also collected at this point. The student perception questionnaire was handed out immediately after the post-test. Three months later, the same MCT was repeated during a compulsory lecture at campus, within the normal teaching hours. The test administration was overseen by one of the authors and standardised across the full-time and part time tracks.

### Traditional teaching method

Students randomised to the TT group received TT delivered by a lecturer who was blinded to the content of the MCT. The lecturer had five years of teaching experience and had repeatedly taught the subject in previous semesters. The thematic structure of the lecture was aligned with the sub-topics in the JS group and aimed to *describe and explain the anatomical structure and function of central elements* in accordance with the course curriculum. The lecture lasted for 105 min, including a 15-minute break between two 45-minute sessions (Table 1). The lecture was given in Norwegian, in an appropriately sized classroom, using a PowerPoint presentation containing 17 slides with text and illustrations. At the beginning of the lecture, students were informed that they were free to ask questions during the lecture. During the lecture, several questions were asked by students. None of the questions could be identified as arising as a direct consequence of the questionnaire. In addition, the students were given the opportunity to study enlarged physical anatomy models of the eye and ear during the break.

### Jigsaw teaching method

Concurrently, the students randomised to JS were further randomised into primary groups, in which they spent five minutes to distribute the sub-topics among the group members, forming the basis for who would become expert in each sub-topic. Immediately after this, the students reorganised into the new expert groups, now consisting of one representative from each of the primary groups. Each expert group collaborated about deepening their understanding on their respective common sub-topic, preparing a 12-minute presentation to take back to their respective primary groups. The students were instructed to address the relevant curriculum for their respective sub-topic but were free to choose their presentation format. During the work in the expert groups, the students managed their time independently, while the lecturer provided facilitation, guidance and clarification. The students had unrestricted access to learning resources in both digital and print formats, including the course textbook and printed materials, a subscription-based online anatomy learning platform and all other publicly accessible online content.

In the next phase of the procedure, the sub-topic experts relocated to their initial primary groups, where they each held a 12-minute presentation. The timing and transition between sub-topic presentations within the primary groups were moderated by a lecturer with experience from JS teaching.

Immediately after the allocation of the expert groups in one of the consecutive tracks, one participant dropped out. As a result, the size of one expert group differed from the others. Consequently, the primary group to which the dropout had been assigned was given a 12-minute break during the presentation period, when the other primary groups covered the specific sub-topic originally allocated to the dropout. Thereafter, the missing sub-topic was covered by an expert from another primary group as an immediate 12-minute extension of the 60 min of teaching. By doing so, all participants received teaching covering all subtopics.

### Ethics

The study was approved by the Norwegian Agency for Shared Services in Education and Research (ref. no.: 841608), ensuring compliance with ethical standards and data management protocols required for academic research. In addition, the study adhered to the Western University of Applied Sciences’ general guidelines for the collection, handling and storage of personal data. Participants were informed that participation was voluntary and that they could withdraw their consent at any time without providing a reason. They were also assured that abstaining from participation would not affect their academic standing or progression. Lastly, regardless of participation, all students were informed that there would be a regular lecture covering the same content later in the semester.

### Statistical analyses

We report descriptive data using means, standard deviations, ranges, counts or percentages, as appropriate.

For the MCT data, we used a longitudinal model fitted using generalised least squares. The model includes separate mean parameters for each combination of timepoint and group (traditional vs. Jigsaw), except for the pre-randomisation baseline timepoint, where, to properly take into account any (chance) baseline group differences, a common mean parameter was used [28, 29]. The model had an unstructured covariance matrix, i.e., allowing for different variances at each timepoint and different correlations between pairs of timepoints. Using the estimated model means, we report both group differences at each post-baseline timepoint and group-specific changes between pairs of timepoints.

Differences in student perceptions between the two groups were compared using an exact Wilcoxon–Mann–Whitney test, and the reported *P*-values are mid-*P*-values [30].

One person dropped out from the study before completing the learning activity and was excluded from all analyses. There were otherwise very little missing data and only in the MCT scores. For analyses with missing data, we report the number of students/observations each analysis is based on. The use of a longitudinal model for the MCT scores also makes it possible to include data from *all* students, even if some of them have missing data at follow-up.

All statistical tests are two-tailed, *P*-values ≤ 0.05 are characterised as statistically significant, and all confidence intervals (CIs) are 95% CIs. The data were analysed using R version 4.5.0 [31] with the packages ‘nlme’ version 3.1–168 [32], ‘marginaleffects’ version 0.25.1 [33] and ‘coin’ version 1.4-3 [34].

## Results

After excluding the student who dropped out before completing the learning activity, a total of 47 participants were included, 23 in the TT group and 24 in the JS group. See Fig. 1 for a flowchart of the study participants. Six participants were lost to follow-up at three months, four in the TT group and two in the JS group.

### Overall change

**Fig. 2:**
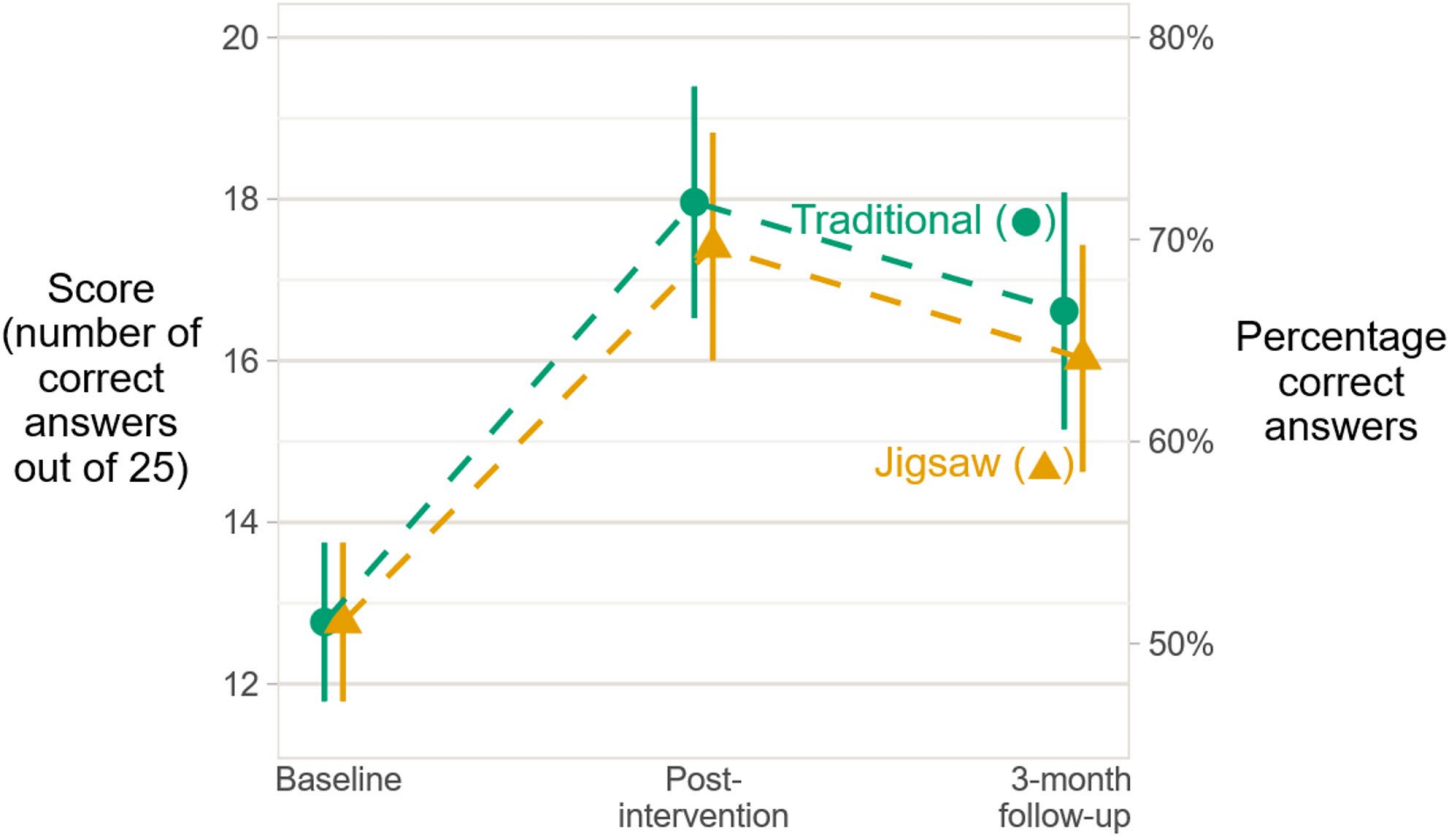
Estimated mean scores for students following a traditional or a Jigsaw-based learning activity (*n* = 47 students and 135 scores). The estimates are based on a longitudinal regression model. The vertical lines show 95% confidence intervals.

As shown in Fig. 2; Table 2, from baseline to post-intervention, both groups showed a large and similar overall improvement in scores. The mean improvement was 5.2 points (CI: 3.9–6.5, *p* < 0.001) for the TT group and 4.6 points (CI: 3.4–5.9, *p* < 0.001) for the JS group (*p* = 0.54 for TT vs. JS). 

**Table 2:** Mean scores* for each teaching method at the three time points, and comparison of mean score between the two teaching methods (*n* = 47 students** and 135 scores)

	Traditional		Jigsaw		Diff. (Jigsaw − traditional)		
	Est.	95% CI	Est.	95% CI	Est.	95% CI	*P*-value
Baseline	12.8	11.8 to 13.8	12.8	11.8 to 13.8	–	–	–
Post-intervention	18.0	16.5 to 19.4	17.4	16.0 to 18.8	−0.5	−2.3 to 1.2	0.54
3-month follow-up	16.6	15.1 to 18.1	16.0	14.6 to 17.4	−0.6	−2.4 to 1.2	0.52

*Abbreviations: Est.* estimate, *Diff.* difference, *CI* confidence interval

* Based on a longitudinal regression model adjusting for any baseline differences and taking into account missing data

** Total number of students. At the 3-month follow-up, there were 6 missing participants

From post-intervention to the 3-month follow-up, there was a mean deterioration of 1.3 points (CI: 0.1–2.6, *p* < 0.03) for the TT group and 1.4 points (CI: 0.3–2.5, *p* < 0.02) for the JS group.

Finally, at the 3-month follow-up, both groups had reached a similar mean improvement from baseline, 3.8 points (CI: 2.5–5.2, *p* < 0.001) for the TT group and 3.3 points (CI: 2.0–4.5, *p* < 0.001) for the JS group (*p* = 0.52 for TT vs. JS).

The estimated (residual) standard deviations at baseline, post-intervention and follow-up were 3.4, 3.9 and 3.8, respectively. The estimated correlations were: baseline vs. post-intervention: 0.60; baseline vs. follow-up: 0.63; post-intervention vs. follow-up: 0.75.

### Individual change

**Fig. 3:**
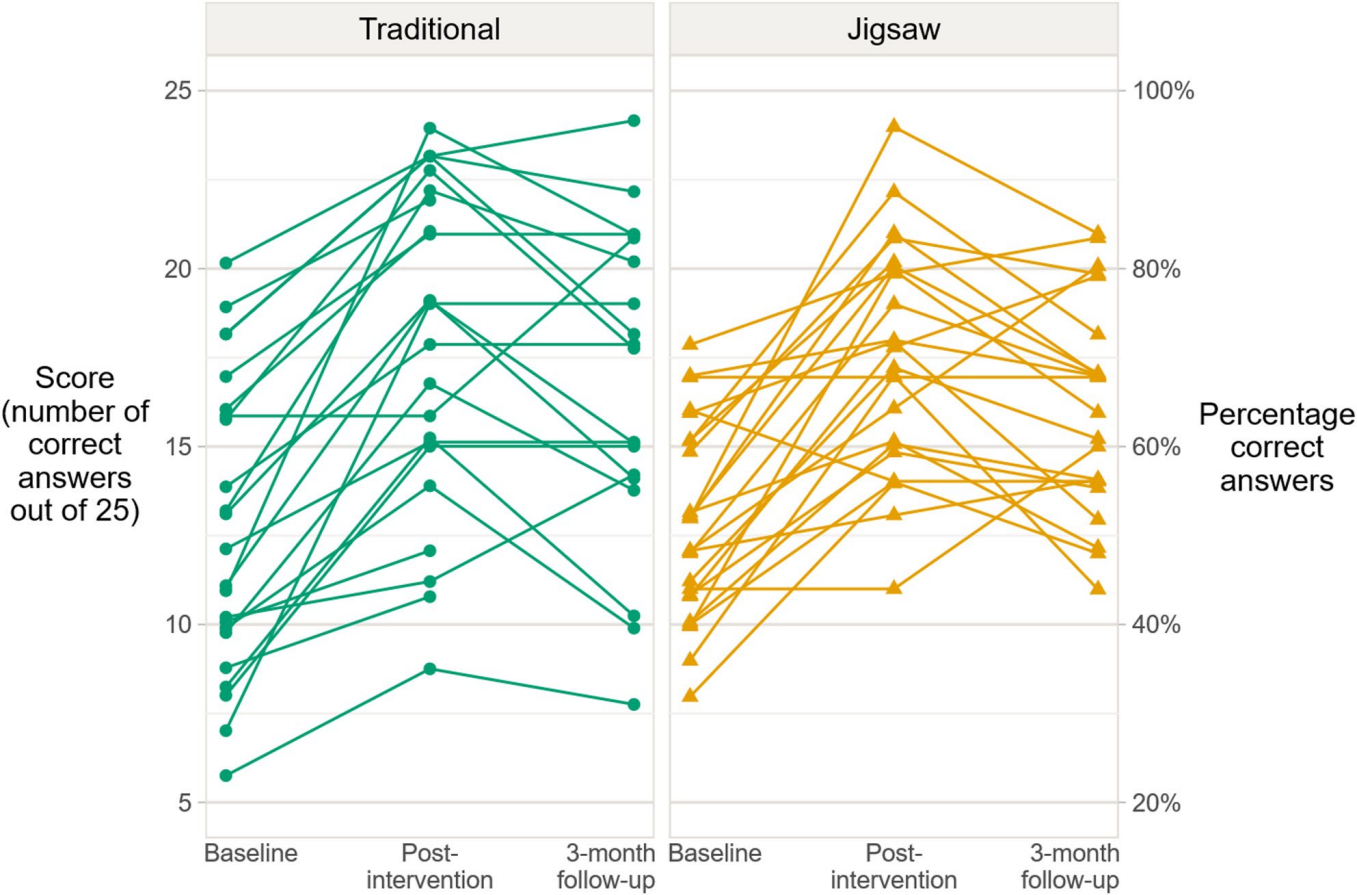
Individual scores for students following a traditional or a Jigsaw-based teaching activity (*n* = 47 students and 135 scores). At the 3-month follow-up, six students had missing data. The scores were integer-valued, but have been randomly shifted up or down by a small amount (less than a ¼ of a score point) in the figure to reduce the effect of overplotting

The individual changes were similar in the two groups. Most students (91%) had a (usually large) score improvement from baseline to post-intervention, but from post-intervention to the 3-month follow-up, most students had a (usually smaller) decline (63%) or no change (17%). The majority (83%) ended up with a higher score than at baseline. See Fig. 3; Table 3 for details.

**Table 3:** Direction of change in score for individual students (*n* = 47 students)

Time period	*n*	Reduced score	No change	Improved score
Baseline → post-intervention	47	2%	6%	91%
Post-intervention → follow-up	41	63%	17%	20%
Baseline → follow-up	41	10%	7%	83%

*Abbreviations: n* number of students with scores at both time points

### Student perceptions

**Fig. 4:**
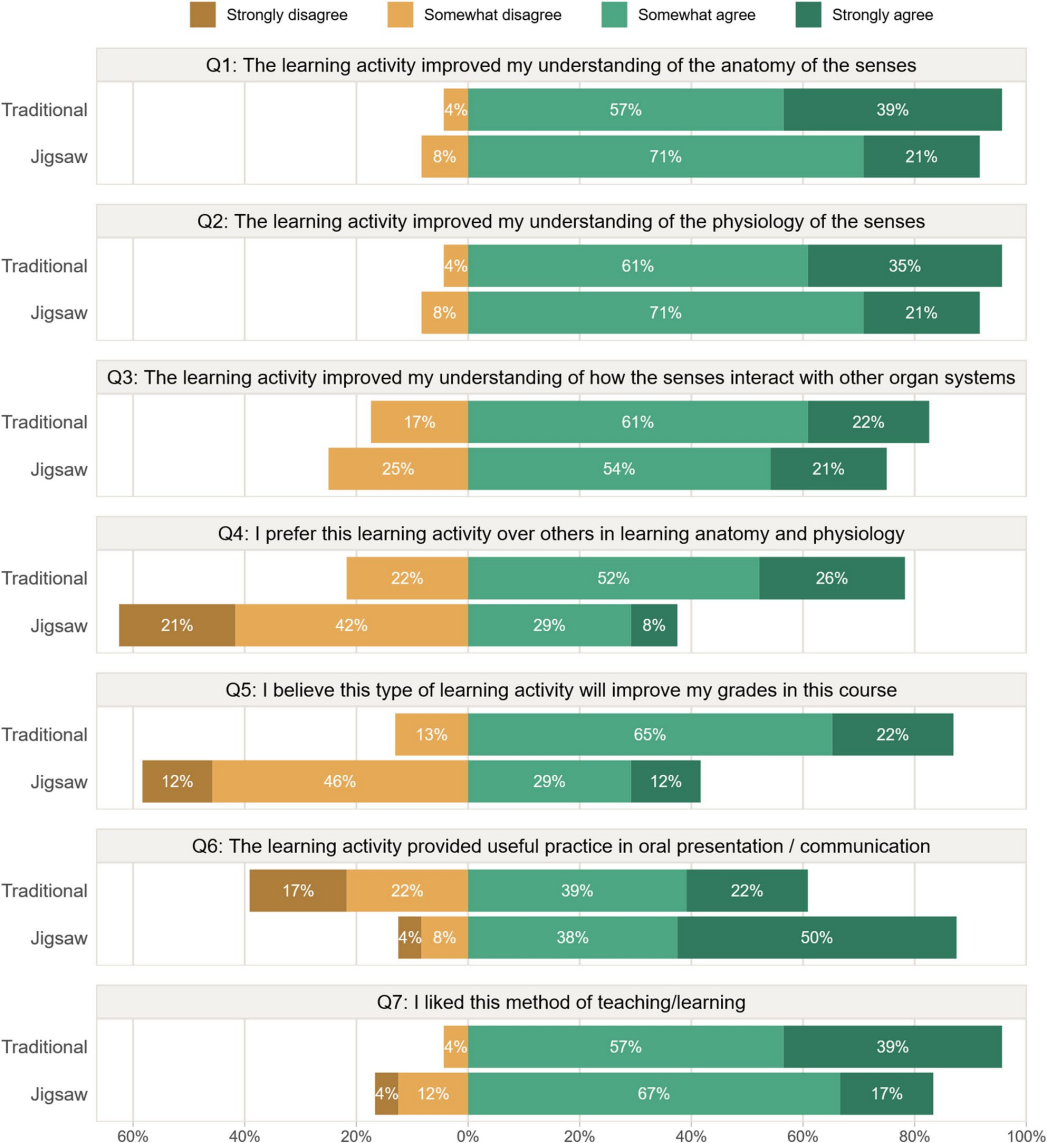
The students’ agreement on various statements on the learning experience, stratified by type of learning activity (*n* = 47 students). Corresponding *P*-values for comparing the two groups are listed in Table 4

Figure 4 shows detailed responses to each statement on the students’ perceptions of the teaching activities, while Table 4 summarises the findings and compares the two groups.

**Table 4:** The proportion of participants agreeing (somewhat or strongly) with each statement (*n* = 47)

Statement*	Traditional	Jigsaw	*P*-value**
Q1: Improved understanding of anatomy	96%	92%	0.16
Q2: Improved understanding of physiology	96%	92%	0.25
Q3: Improved understanding of interaction with other systems	83%	75%	0.67
Q4: Prefer this learning activity over others	78%	38%	0.002
Q5: Believe this learning activity will improve grades	87%	42%	0.004
Q6: The activity provided useful practice in oral presentation	61%	88%	0.02
Q7: Liked the method of teaching/learning	96%	83%	0.05

* Abbreviated text. See Fig. 4 for a complete description of each statement

** The *P*-value is calculated based on all four response categories (strongly disagree, somewhat disagree, somewhat agree, strongly agree)

Overall, the students’ perceptions on how the teaching activities improved their understanding of the curriculum (Q1–Q3) were similar in the two groups. However, a larger proportion of students in the TT group than in the JS group preferred the given activity over other activities (Q4, 78% vs. 38%, *p* = 0.002) or believed that the activity would improve their grades (Q5, 87% vs. 42%, *p* = 0.004).

On the other hand, proportionally *fewer* students in the TT group than in the JS group thought that the activity provided useful practice in oral presentation / communication (Q6, 61% vs. 88%, *p* = 0.02). Overall, both groups liked the activity, but slightly more students in the TT group did so (Q7, 96% vs. 83%, *p* = 0.05).

## Discussion

### Summary

This randomised, controlled trial compared the effects of TT and JS in higher education students’ knowledge of the anatomy and physiology of the sensory apparatus. At both the group level and the individual level, both methods resulted in improved student scores in the short and long term. At no timepoint were there any statistically significant differences in knowledge between the two groups.

There were some differences in the two groups’ perceptions of the activities, but the only apparent advantage of JS was that more students believed it provided useful practice in oral presentation / communication.

### Student knowledge

#### Short-term effects

The reported JS short-term (immediately after teaching) mean knowledge improvements of JS in the present study are in line with the findings in a non-randomised study by Oakes et al. [17], which reported that the students who participated in a two-hour JS workshop during an abdominal anatomy and physiology course improved their short-term scores on a workshop quiz (*p* < 0.01). They are also in line with the non-randomised study by Lalit and Piplani [16], which reported that first-year medical students scored higher (*p* < 0.001) after participating in a Jigsaw activity than on a pre-Jigsaw test. Note that this study used a different post-test than the one used in the pre-test, so the difficulty of the tests may not have been identical. Our results partly contrast with Chandel et al. [19], which reported statistically significant improvements from pre- to post-test in only one out of four modules (*p* = 0.026; the *p*-values for the other three modules being > 0.21).

Our results add to these previous findings by strengthening the evidence that JS provides short-term knowledge gains within the field of anatomy and physiology. The estimated short-term effect in our study was a mean increase of approximately 4.6 more correct answers on the 25-question MCT, which corresponds to an absolute increase of approximately 18% points if the scale is converted to a 0–100% scale.

The present study did not demonstrate any statistically significant short-term differences in learning between JS and TT. Additionally, the 95% confidence interval for the difference was narrow (− 2.3 to 1.2), thereby excluding any large differences in the effect of the two teaching methods on short-term learning outcomes. These findings contrast to Ziyai and Dikmen [18] who performed a randomised quasi-experimental study and demonstrated that the JS group performed better (*p* < 0.01) post-experiment on a 20-item knowledge test on arterial blood pressure. However, the educational content of the two interventions differed considerably. For example, the topics covered by the JS expert groups (but not the TT group) was based on what students did not understand on a quiz taken before the formation of the expert groups. The authors also did not report on pre-scores or time used on TT and JS, which limits the interpretation of the results.

The short-term similarities of JS and TT also partly contrast to the findings in the controlled crossover study by Chandel et al. [19]. They demonstrated that JS students performed better than TT students on 25-item post-intervention MCTs for three out of four anatomy and physiology modules (*p* < 0.001, < 0.001, 0.041 and 0.285), while there were no differences in the corresponding pre-intervention MCTs. The discrepancy between the reported short-term differences in Chandel et al. [19] and in the present study may reflect differences in the methodological approaches. In the present study, JS and TT were completed in single two- and three-hour sessions, whereas Chandel et al. [19] implemented the teaching over three separate one-hour sessions with unspecified intervals between the sessions. Further, it is possible that the topics in the two studies had different levels of difficulty and implied cognitive demands. Berger & Hänze [35] has demonstrated that JS may be better suited to less challenging topics.

#### Long-term effects

Our data show that compared to the baseline results, JS resulted in improved knowledge up to three months after baseline. This strengthens the evidence that JS causes long-term learning in anatomy and physiology teaching. However, the results partly contrast with Chandel [19], who reported that JS led to higher scores after four weeks than at baseline in only one out of four modules (*p* = 0.028; the *p*-values for the other three modules being > 0.36).

The present study did not detect differences between JS and TT at the three-month follow-up. In contrast, Oakes et al. [17] administered an anatomy end-of-semester exam (ESE) and found that JS led to higher overall ESE scores compared to a control group in one of two cohorts (*p =* 0.025). However, when analysing the subset of the ESE questions covering the specific topic taught through JS, no differences were observed. However, it is important to note that Oakes et al. [17] compared TT alone (control) with TT combined with JS (intervention group).

The absence of long-term JS and TT differences contrast to Chandel et al. [19], which demonstrated that JS led to higher scores in a retention test compared to TT in three of four topics (*p* < 0.001). Our study conducted a follow-up MCT three months after the intervention, while Chandel et al. [19] performed a retention test four weeks after, so the findings are not directly comparable. While the findings reported by Chandel et al. [19] are noteworthy, the discrepancy between our results and theirs suggests that the effect of JS compared to TT on knowledge is influenced by the implementation, level of difficulty of the curriculum and/or the testing strategy.

#### Effects at the individual level

We have not found any studies examining the effect of JS on learning at the individual level. In our study, most students in both the JS and TT group followed a similar pattern: a large improvement from baseline to post-intervention followed by a smaller decrease to the three-month follow-up. Only a few individuals showed no improvement from baseline to post-intervention or to follow-up. This suggests that the short- and long-term learning effect of both JS and TT is generally consistent across students.

### Student perceptions

Our results show that JS participants had a generally positive perception of the effect JS had on their understanding of the curriculum (Q1–Q3). Similar positive perceptions are reported for JS in several prior studies [14, 17, 19, 20]. We found no difference between the JS and TT students’ perceptions of the effect of the teaching procedures on their understanding.

In line with Oakes et al. [17], Bhandari et al. [14] and Soundarya et al. [20], a large proportion (88%) of the JS participants agreed that JS provided useful practice in oral presentation / communication.

A much larger majority (87%) of the TT participants than the JS participants (42%) believed that the learning activity would improve their grades. These differences stand in contrast to the actual learning outcomes as measured by the MCT, where no large or statistically significant differences were found between the groups. This demonstrates that students’ impressions of what works do not necessarily align with what actually improves learning [8]. The disparity may further indicate that evaluation strategies that rely solely on student perceptions can be misleading.

In our study, 78% of the TT participants preferred their assigned learning activity over other methods, while only 38% of the JS participants did so. Deslauries et al. [8] argue that good teachers can positively influence students’ feelings of learning, and students therefore may favour TT over active teaching strategies even when they learn more from the latter.

### Strengths and limitations

A strength of this study is the randomisation to TT and JS, as well as to the primary groups in JS. Internal validity was further strengthened by statistical adjustments for chance baseline differences between the groups. Both the TT and JS lecturers had prior experience with the respective teaching methods. This is important, as teacher literacy may impact the outcome of JS teaching [15].

The systematic development of the MCT in accordance with established frameworks strengthen both the face and content validity and adds credibility to the outcome measures. The inclusion of 25 questions in the MCT allowed for a broad coverage of the curriculum content and ensured consistency across sub-topic content areas.

The low drop-out rate and the relatively narrow confidence intervals add to the credibility of the findings. When considered alongside the substantial and consistent group level score improvements from baseline to post-intervention and follow-up, the results demonstrate statistical robustness. The results on the individual level show a consistent pattern across participants, contributing to a more comprehensive understanding of the MCT outcomes.

Limitations include a relatively low inclusion rate that may have introduced participation bias. Concerning the practical execution of the experiment, the JS group was allocated more total time (165 min) for the teaching procedure than the TT group (105 min), possibly influencing the comparability of the two approaches. However, the JS participants were only exposed to 48 min of structured peer teaching, supplemented by their own contribution (12 min) within primary groups. In addition, they engaged in clarifying tasks, planning and active study of the curriculum; therefore, it is reasonable that somewhat more time is allocated to JS. In contrast, participants in the TT spent less time on the learning activity in total but were exposed to 90 min of structured teaching as passive receivers. These fundamental differences in the nature of the teaching methods mean that total time spent on the learning activity may not be a completely valid indicator of learning exposure. It is still noteworthy, though, that even with more time allocated to JS, we did not observe improved knowledge acquisition for JS relative to TT.

By administering the same MCT at all three timepoints (pre, post and follow-up), we ensured consistent knowledge assessment. Nevertheless, there is a potential for priming, as exposure to a MCT before a learning procedure may activate a deeper processing of the information related to the test questions [36], and participating in a test can itself be a learning activity [37]. The use of the same MCT therefore increases the risk of measuring the combined effect of the MCT and JS, rather than isolating the effect of JS alone. This risk may be particularly relevant from the pre- to the post-test, as Little and Bjork [36] has demonstrated that exposure to a multiple-choice test before reading improved performance on a 48-hour retention test.

On the other hand, using a different MCT at all three timepoints also entails validity problems. For example, a score increase from the pre-test to the post-test may be the results of reduced difficulty of the post-test rather than of actual learning. It is difficult to construct three separate tests that all have the same difficulty and at the same time test the students’ knowledge of a specific curriculum.

Wood [38] examined the effect of prior exposure of questions on MCTs. They found that after repeated test administration, the examinees’ scores on reused questions increased, but their results on non-reused (novel) questions also increased by the same amount. They concluded that examinees did ‘not appear to be advantaged by seeing reused questions’. In our study, we also had additional safeguards, in that participants were not informed that the same test would be repeated, and no feedback or review of answers was provided after test administration at any timepoint.

Still, other approaches to the study design are possible. For example, we could have added an additional control group with no exposure of the pre-test. This would have allowed us to isolate the effect of the pre-test on the post-test scores. We could also have developed two parallel tests, A and B, and randomised half the students to get test A as a pre-test, the other half to get test B, and switch the tests around for the post-test. Then all students would get a MCT with ‘novel’ questions at the post-test, and it would be possible to measure and adjust for any difference in test difficulty between tests A and B. (For the three-month follow-up, we would need an additional test and divide the students into three groups.) Yet, these approaches are much more complex and logistically challenging, and we believe the benefits of using them is modest.

It should be noted that the *student perceptions* questionnaire was administered immediately after the post-intervention MCT. Thus, it cannot be ruled out that the experience of completing the MCT may have influenced the students’ responses to the questionnaire.

As with all pedagogical research, challenges remain with reproducibility of real-world teaching environments. This was present in our study as the experiment was conducted over two sessions, potentially affecting standardisation and experimental control, particularly in the TT sessions. Nevertheless, research in actual classrooms possesses high ecological validity [39] and can give valuable insights into real-world situations.

The sensory apparatus represents only a minor part of the course curriculum. However, the topic is widely covered within comparable courses across higher education disciplines with comparable level of difficulty (e.g., nursing, paramedicine and sports science), and the results may thus be generalisable at least to such contexts.

## Conclusion

This study found that both the Jigsaw method and traditional lectures effectively improved students’ knowledge in anatomy and physiology. Despite a preference for traditional teaching among the students, there were no significant differences in knowledge acquisition or retention between the two methods.

## Data Availability

An anonymised version of the dataset for this study is available upon reasonable request from the corresponding author.

## Abbreviations

JS: Jigsaw
TT: Traditional teaching
MCT: Multiple-choice test
ESE: End of semester exam
